# Myeloid-Specific Blockade of Notch Signaling Attenuates Choroidal Neovascularization through Compromised Macrophage Infiltration and Polarization in Mice

**DOI:** 10.1038/srep28617

**Published:** 2016-06-24

**Authors:** Guo-Rui Dou, Na Li, Tian-Fang Chang, Ping Zhang, Xiang Gao, Xian-Chun Yan, Liang Liang, Hua Han, Yu-Sheng Wang

**Affiliations:** 1 Department of Ophthalmology, Xijing Hospital, Fourth Military Medical University, Xi’an 710032, China; 2 Department of Medical Genetics and Developmental Biology, Fourth Military Medical University, Xi’an 710032, China; 3 Department of Biochemistry and Molecular Biology, Fourth Military Medical University, Xi’an 710032, China

## Abstract

Macrophages have been recognized as an important inflammatory component in choroidal neovascularization (CNV). However, it is unclear how these cells are activated and polarized, how they affect angiogenesis and what the underlining mechanisms are during CNV. Notch signaling has been implicated in macrophage activation. Previously we have shown that inducible disruption of RBP-J, the critical transcription factor of Notch signaling, in adult mice results in enhanced CNV, but it is unclear what is the role of macrophage-specific Notch signaling in the development of CNV. In the current study, by using the myeloid specific RBP-J knockout mouse model combined with the laser-induced CNV model, we show that disruption of Notch signaling in macrophages displayed attenuated CNV growth, reduced macrophage infiltration and activation, and alleviated angiogenic response after laser induction. The inhibition of CNV occurred with reduced expression of VEGF and TNF-α in infiltrating inflammatory macrophages in myeloid specific RBP-J knockout mice. These changes might result in direct inhibition of EC lumen formation, as shown in an *in vitro* study. Therefore, clinical intervention of Notch signaling in CNV needs to pinpoint myeloid lineage to avoid the counteractive effects of global inhibition.

Choroidal neovascularization (CNV), the immature neo-vessels arising from the original chorio-capillaries into the subretinal space and toward the outer retina, accounts for severe visual impairment in several ocular diseases, such as wet form of age-related macular degeneration (AMD) and pathologic myopia^1^. The precise mechanisms of this fibrovascular formation in CNV development are largely unknown, but it is a general consensus that progressive inflammatory cascades and macrophage infiltration contribute to retinal pigment epithelium (RPE)-Bruch’s membrane damage in AMD and associated CNV^2^. Despite the function and phenotype of macrophages conditioned by signals within the CNV microenvironment that appear controversial and are still under intensive investigation, the accumulation of myeloid cells, especially macrophages are thought to be central to the initiation and progression of this disease^3^,^4^. Macrophages involved in angiogenesis has been reported to be key players as an abundant source of inflammatory and angiogenic factors, notably vascular endothelial growth factor (VEGF), interleukin (IL)-1β, tumor necrosis factor (TNF)-α, and IL-6^5^. Moreover, the paradigm of M1 versus M2 macrophages has been studied with respect to angiogenesis in recent years^6^. Classical activation generates M1 macrophages that have pro-inflammatory functions, whereas alternatively activated M2 macrophages confer responses related to promoting wound healing and angiogenic responses. It has been reported in a pilot study that macrophage polarization in the macular retina and choroid of AMD and non-AMD subjects were detected, suggesting a potential role of the pathological shift of macrophage polarization in AMD pathogenesis^7^. Recently, a study by He and Marneros^8^ revealed that infiltrating macrophages in laser-induced CNV lesions are M2 macrophages with strong upregulation of the prototypic M2 type marker arginase (Arg)-1. M2 macrophages phenotype that is elicited by its phagocytosis of damaged RPE components is assumed as a potential early angiogenic driver in laser-induced CNV model^9^. A recent study also indicates that M2 macrophages, rather than M1, play an important role in promoting retinal pathological neovascularization probably by producing secreted factors^10^. It has been reported that certain signals or agent such as CD200R, Doxycycline, and Rho-associated kinase signaling may act as underlying candidates in targeted intervention on macrophages^11^,^12^. Thus, it is critical to elucidate the molecular mechanisms involved in modulating the phenotype and function of macrophages before clinical manipulation of early events that trigger CNV.

The Notch signaling pathway plays a crucial role in specifying cellular fates on several types of cells including myeloid lineage and macrophages by regulating communication between adjacent cells^13^,^14^,^15^,^16^. Triggered by ligands expressed on neighboring cells, Notch receptors release the Notch intracellular domain (NICD) by a series of enzymes-catalyzed proteolytic reactions involving γ-secretase. NICD then translocates into the nucleus and transactivates the recombination signal binding protein Jκ (RBP-J) to initiate the expression of downstream effectors such as the Hairy and enhancer of split (Hes) family proteins^17^. Resting macrophages express all four types of Notch receptors^13^,^18^ and activated macrophages selectively further increase Notch 1 expression^13^. It has been implicated that Notch signaling plays an important role in myeloid cell differentiation^19^ and in the regulation of cytokine expression in mature macrophages^13^,^18^,^20^. For instance, in central nerve system, modulation on Notch signaling in microglia/macrophage regulates pro-inflammatory cytokines secretion and nitric oxide production as well as an increase in phagocytic activity^14^. Notch signaling-regulated macrophages could further participate in diseases pathogenesis under several conditions. Evidence has shown that Notch1 regulates VEGFR-1 and inflammatory cytokines expression in macrophages during wound healing in mice^20^. Notch signaling might not only be required for initial macrophage activation, but also participate in polarization at later inflammatory stages. Our group has provided evidence that Notch inhibition by GSI or RBP-J knockout skews macrophage polarization in tumor, hepatic fibrosis and spinal cord injury^16^,^21^,^22^. Notch1 signaling-dependent macrophage M2b polarization might play a pivotal role in the pathogenesis of systemic lupus erythematosus^23^. However, whether Notch signaling-mediated macrophage phenotype modulation is involved in CNV has been obscure.

Previous studies from Ahmad and our group have suggested that Notch signaling is a key regulator of CNV and thus a molecular target for therapeutic intervention in wet AMD^24^,^25^. Yet the role of Notch signaling in CNV is much more complicated by the findings that Notch directly participates in functional remodeling of different cell types. In angiogenesis, for instance, it has been widely accepted that Notch directs proliferation and specification of endothelial cell^26^. Notch activation in endothelial cells could be triggered by neighboring endothelial cells, and also macrophages as well. Outtz *et al*. have shown that retinal macrophages localize between Dll4-positive tip cells and at vascular branch points, which could be disrupted by myeloid-specific deletion of Notch1^27^. Intriguingly, Notch signaling may play different roles in different cell types involved in CNV. Study from Camelo group have implied that Notch activation on macrophages and ECs could lead to opposing effects in inflammatory neovascularization in CNV^28^. Therefore, how Notch-mediated macrophages influence endothelial function and fibrovascular growth in situations such as CNV need to be further elucidated.

To clarify these questions, in this study, we introduce laser-induced CNV model on myeloid specific deletion Notch signal mouse model (Lyz2-Cre-mediated RBP-J knockout mouse). We show that myeloid-specific RBP-J knockout attenuated CNV growth accompanied with compromised M2 macrophage and inflammation response.

## Result

### Myeloid RBP-J cKO Mice Exhibited Normal Retinal Development

**Figure 1:**
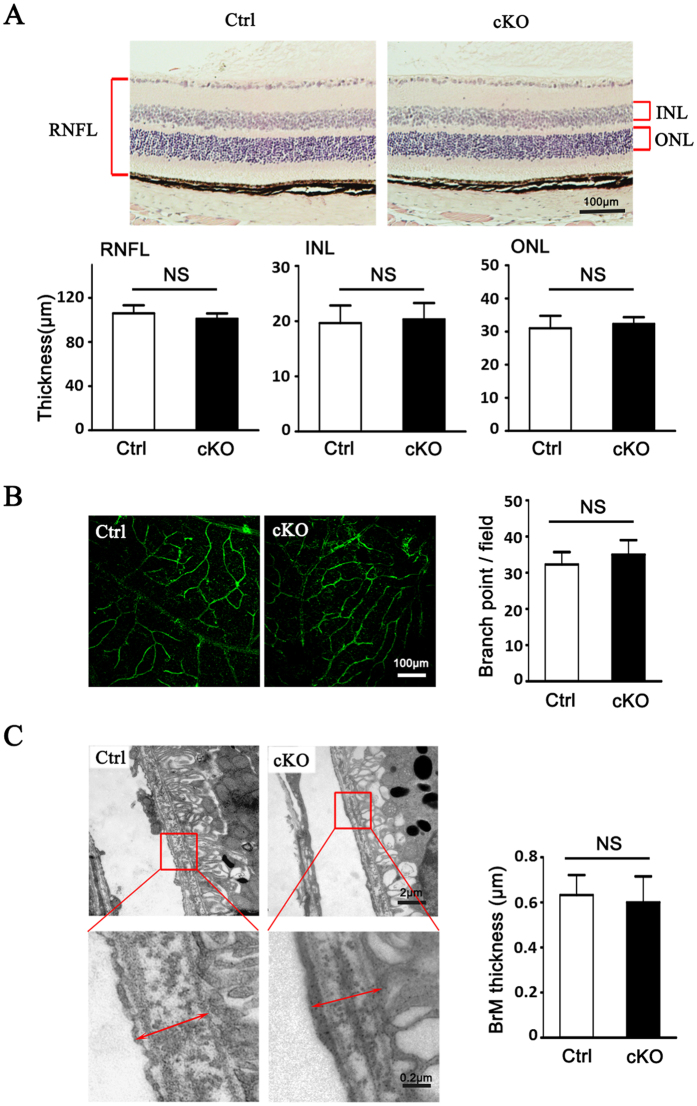
Myeloid specific conditional RBP-J knockout (RBP-J cKO) did not result in significant abnormality in eye. (**A**) Photomicrographs of the RBP-J cKO (cKO) and control (Ctrl) retina showed similar morphology (H&E). Histograms show the thickness of retinal nerve fiber layer (RNFL), inner nuclear layer (INL), and outer nuclear layer (ONL), respectively. Briefly, three sections of retina tissues with optic nerve were randomly selected from per eye for H&E. The thickness was measured and calculated to get the average thickness, and was compared between two groups. (**B**) The retinal blood vessels visualized by lectin staining and quantification of the branch points per field. Three representative microscopic fields were examined in each retina and branch points per field were counted. The average number of branch points was calculated. Histogram indicates the comparison the average number of branch points in two groups. (**C**) Representative transmission electron micrograph of the similar subretinal region of RBP-J cKO and control mice. Briefly, three representative fields were randomly selected from each eye for T.E.M. The average thickness was calculated, and was compared between totally 4 mice per group. Data are presented as mean ± SEM (n = 4 eyes from 4 mice per group). NS, no significance.

Previously, we reported that adult mice with global RBP-J deletion exhibited extensive and excessive neo-blood vessels in the cornea, iris and retina^25^. To determine if RBP-J deletion specifically in myeloid cells induces changes in fundus, we observed the histology changes of eyes from RBP-J cKO mice (Fig. 1). Compared with the control mice, eye sections showed normal architecture with well-defined nuclear layers (GCL, INL and ONL), synaptic layers, intact photoreceptors (inner and outer segments) and the RPE layer (Fig. 1A). The retinal flatmount showed intact vasculatures representing the normal architecture of retinal vessels (Fig. 1B). We did not detect any thickening or drusen in the Bruch’s membrane or patches of choroidal vessels which is typical morphology of retinal degeneration by electron micro-scan (Fig. 1C). These results indicated that myeloid RBP-J cKO did not interfere with retinal development or homeostasis.

### Notch Signaling Activation was Increased during CNV Development

**Figure 2:**
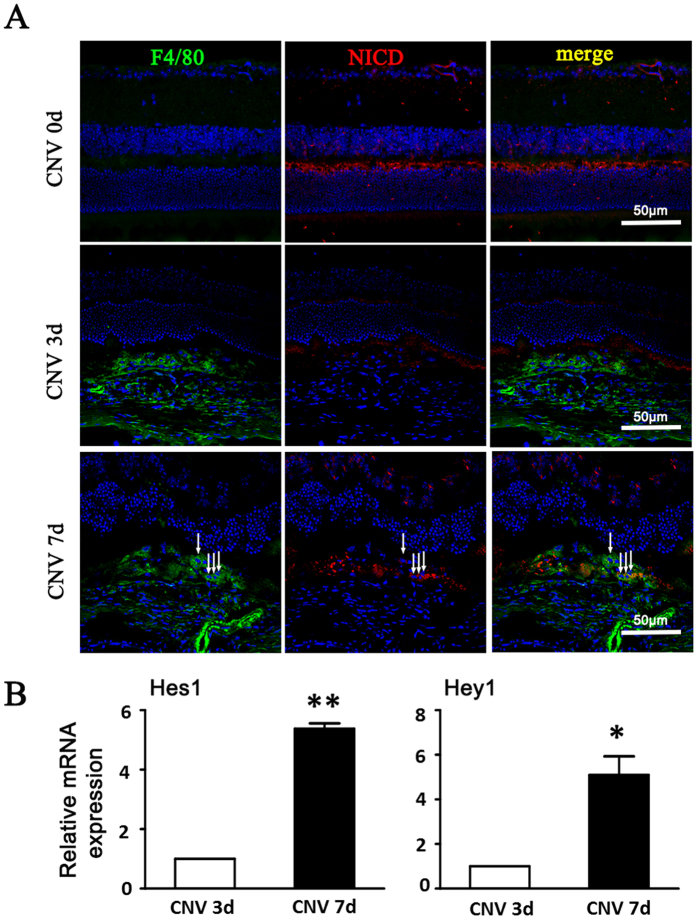
Notch activation in the choroidal tissues of mice with induced CNV. (**A**) The eyes of wild-type C57/B16 mice were subjected to laser-induced CNV. Eye cups were collected at the indicated time points after laser treatment, and were stained with anti-F4/80 and anti-NICD. Arrows indicated NICD^+^ macrophages. (**B**) The two eyes of one mouse were used as one set of RNA retraction. The relative mRNA level of Hes1 and Hey1 was determined by using qRT-PCR, with β-actin as an internal reference control. Each individual experiment was repeated at least three times. Data are presented as mean ± SEM (n = five mice per group). *P < 0.05, **P < 0.01.

To determine the involvement of myeloid Notch signaling in CNV development, eye sections were stained with active NICD antibody at different time points before and after laser coagulation in wild-type C57/BL6 mice (Fig. 2). Low level of active NICD expression was detected in the neuronal retina in the inner plexiform layer, outer plexiform layer and in ganglion cells before laser coagulation (Fig. 2A). Three days after laser treatment, the level of active NICD increased mildly in CNV lesions and the NICD levels reached significantly high level till day 7 (Fig. 2A). Dual staining of F4/80 and active NICD showed that Notch signal was also active in the infiltrating F4/80+ subretinal macrophages/microglia (white arrow) (Fig. 2A). Meanwhile, we also detected expression of Notch downstream gene Hes1 and Hey1. The mRNA level of Hes1 and Hey1 were greatly upregulated on day 7 after laser treatment (Fig. 2B).

### Genetic Deletion of RBP-J in Myeloid Cells Reduces CNV Lesion Size

**Figure 3:**
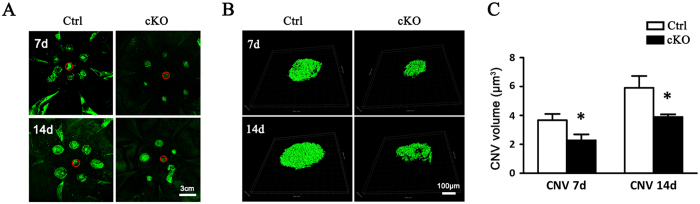
RBP-J cKO mice exhibited reduced CNV severity. RBP-J cKO and control mice were subjected to laser coagulation with six laser burns in one eye (six mice for each group). Choroidal tissues were flat-mounted and stained with FITC-isolectin B4 7 and 14 days later, and examined under a laser-scanning confocal microscope (**A**), followed by reconstruction of the CNV lesions (**B**). The CNV volumes were compared between the two groups (**C**). The optic nerve head are labeled with red circle. Data are presented as mean ± SEM of six RPE/choroid from six mice (six laser burns in each RPE/choroid). *P < 0.05.

To specifically test the role of Notch signaling in macrophages in CNV, we blocked Notch signaling by using myeloid-specific RBP-J knockout mice. The control mice and the RBP-J cKO mice were subjected to laser-induced CNV, and the CNV lesions of the mice were assessed after photocoagulation on day 7 and 14 by scleral-choroidal flatmount stained with isolectin B4 (Fig. 3). We noticed that on day 7 and day 14, there was a significant reduction in CNV area and lesion volume in the RBP-J cKO mice when compared to the control counterparts (P < 0.05) (Fig. 3A,B). The result showed that fiber-vascular formations in the RBP-J cKO mice were growing constantly slower than that of the control mice (Fig. 3C), suggesting that myeloid specific RBP-J knockout attenuated CNV growth.

### Disruption of RBP-J in Myeloid Cells Reduced Retinal Immune Cell Infiltration

**Figure 4:**
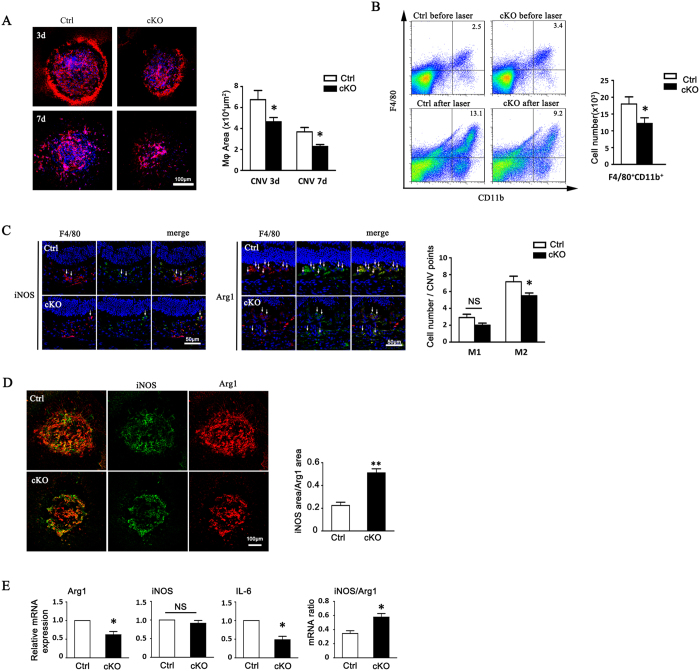
Myeloid specific RBP-J deficiency decreased macrophage infiltration and M2 macrophage polarization in CNV lesions. (**A**) RBP-J cKO and control mice were subjected to laser coagulation. Choroidal tissues were flat-mounted and stained with anti-F4/80 as indicated time points. Macrophage infiltration area was represented as pixels and the average pixels per CNV lesion were calculated. Histogram shows the comparisons on the average area of macrophage infiltration in five mice per group. (**B**) Two eyes of one mouse were adopted as one set to prepare the single cell suspensions from RPE-choroidal tissues in (**A**) at day 3 after laser injury, and analyzed by flow cytometry. The numbers of F4/80^+^CD11b^+^ cells were compared between the two groups (five mice per group). (**C**) Retinal tissues of mice in (**A**) were immunolabeled with F4/80 and Arg1 or F4/80 and iNOS at day 3 after injury. Arrow indicates Arg1^+^ macrophages or iNOS^+^ macrophages. Three representative images per lesion were randomly selected from three biggest CNV lesions in each eye for cell count, and the average number of F4/80^+^Arg1^+^ or F4/80^+^iNOS^+^ macrophages was calculated and compared in five eyes per group. (**D**) Flat-mounted choroidal tissues of mice in (**C**) were immunolabeled with Arg1 and iNOS. The total pixels of iNOS and Arg1 were measured to calculate the ratio of M1/M2. The ratio of M1/M2 was compared between two groups (five eyes per group). (**E**) Total RNA was prepared from choroidal lysates of RBP-J cKO and control mice at day 3 after laser injury. Two eyes of one mouse were adopted to prepare the RNA retraction for qPCR. The mRNA level of Arg1, iNOS, and IL-6 determined with qRT-PCR, with β-actin as an internal reference control. Each individual experiment was repeated at least three times. The expressions of Arg1, iNOS, IL-6, and the ratio of iNOS/Arg1 were compared. Data are presented as mean ± SEM of five eyes or mice per group. *P < 0.05. NS, no significance.

To investigate whether reduced CNV in RBP-J cKO mice was related to macrophage infiltration, we examined macrophage/microglia in CNV lesions by both immunostaining and flow cytometry (Fig. 4). A representation of macrophage distribution around individual laser lesions at 3- and 7-days post laser injury is shown in Fig. 4A. F4/80+ cells recognized as infiltrating macrophage/microglia aggregated in CNV lesions to the peak on day 3 post laser coagulation, and was gradually lessened on day 7 (Fig. 4A). At the CNV lesions, control animals displayed a significant higher density of F4/80+ cells compared with the RBP-J cKO mice both on day 3 and day 7 (P < 0.01) (Fig. 4A). Consistent with the immunostaining on flat mounts, flow cytometry analysis indicated that CNV lesions of RBP-J cKO mice had statistically significantly lower numbers of macrophages at 3 days post laser injury, compared with the wild-type eyes (P < 0.05) (Fig. 4B).

### Myeloid-Specific RBP-J Knockout Reduced M2 Macrophage Polarization

We then examined the functional status of local macrophages by immunofluorescence staining of macrophage polarization markers in CNV lesions on day 3 (Fig. 4). The result showed that the number of F4/80+Arg-1+ M2 macrophages decreased in the lesion area of RBP-J cKO mice as compared with that of the control (Fig. 4C). Meanwhile, the numbers of F4/80+iNOS+ macrophages did not have significant decrease in the lesion area of RBP-J cKO mice (Fig. 4C). The ratio of M1/M2 macrophage was increased compared between cKO and control mice by the analysis of iNOS+/Arg1+ macrophages in CNVM (Fig. 4D), showing the shift of polarization in cKO mice. Consistent with the immunofluorescence staining, mRNA expression of Arg-1 in the CNV lesion of RBP-J cKO mice was downregulated and the ratio of iNOS/Arg1 was upregulated (Fig. 4E). We also detected a reduced mRNA expression of IL-6 in RBP-J cKO mice (Fig. 4E). These results suggested that myeloid specific knockout of RBP-J lead to the declining polarization of macrophages to M2-like phenotype.

### Myeloid RBP-J Regulated Cytokine Expression of BMDMs and in CNV Areas

**Figure 5:**
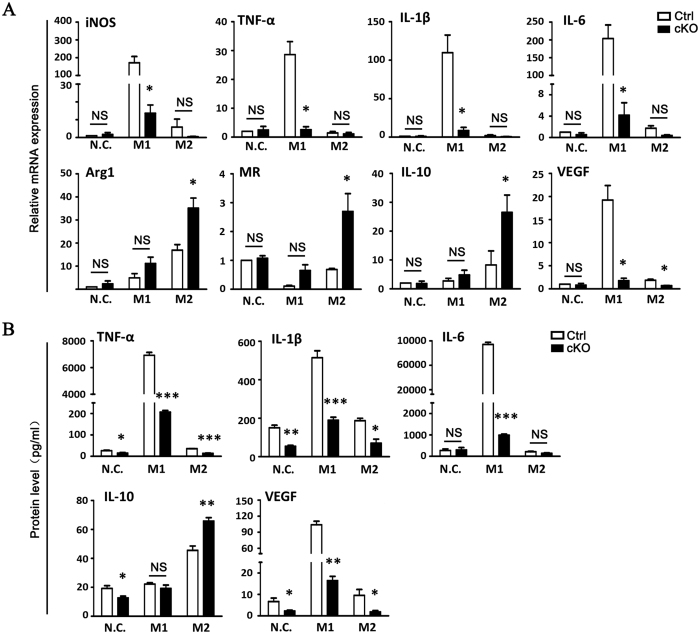
RBP-J-deficient macrophages exhibited a weakened pro-angiogenic phenotype. (**A**) BMDMs were prepared from RBP-J cKO and control mice, and stimulated with PBS (N.C), LPS+IFN-γ (M1) or IL-4 (M2). The expression of iNOS, TNF-α, IL-β, IL-6, VEGF, Arg1, MR and IL-10 mRNA was determined by using qRT-PCR. (**B**) Culture supernatants of cells in (**A**) were collected, and the levels of TNF-α, IL-10, IL-1β, IL-6 and VEGF were determined by using ELISA. Each individual experiment was repeated at least three times. Data are presented as mean ± SEM from three mice per group. *P < 0.05, **P < 0.01, ***P < 0.001. NS, no significance.

Macrophage-related cytokines are believed to play roles in CNV development. We examined the local mRNA expression of cytokines including TNF-α, IL-1β, IL-6, VEGF, and IL-10, as well as iNOS, Arg-1, and MR in BMDMs using quantitative RT-PCR (Fig. 5A). When BMDMs from RBP-J cKO mice were induced to polarize to M1 type with LPS and IFN-γ, most of the molecules examined were downregulated compared with that in the control, but the expression of IL-10 showed no significant change. On the other hand, when BMDMs from RBP-J cKO mice were induced to polarize to M2 type, most of the molecules examined maintained in a low level of expression compared with that of the control, but the expression of Arg-1, MR and IL-10 was upregulated obviously. We further examined the concentration of TNF-α, IL-1β and IL-6, IL-10 and VEGF by using ELISA to determine inflammatory secretion by RBP-J cKO macrophages. The result showed that myeloid specific RBP-J knockout led to lower concentrations of TNF-α, IL-1β, and IL-6. On the contrary, the concentration of IL-10 increased in myeloid specific RBP-J knockout macrophages (Fig. 5B).

**Figure 6:**
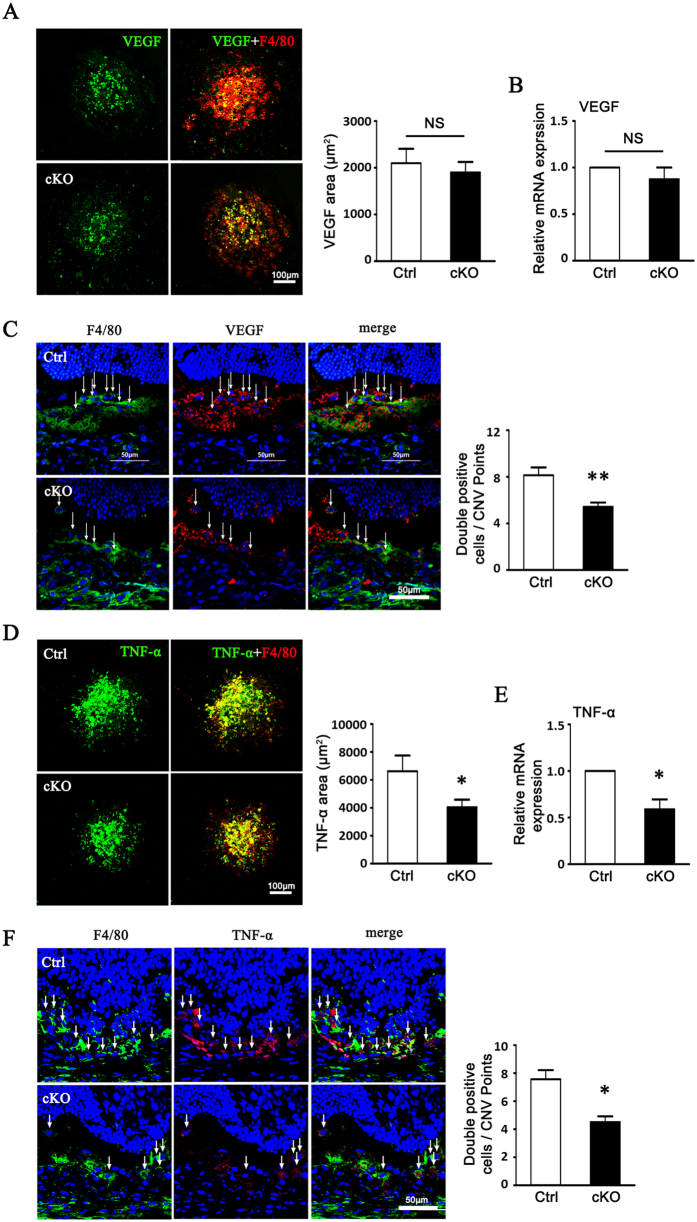
Myeloid VEGF and TNF-α expressions were reduced in CNV lesions of RBP-J cKO mice. (**A**) RBP-J cKO and control mice were subjected to laser coagulation. Choroidal tissues were flat mounted on day 3 and stained with anti-F4/80 and anti-VEGF, and observed under a confocal microscope. The average areas of VEGF expression in 24 randomly selected images were compared between control and RBP-J cKO group. (**B**) Total RNA of two eyes from one mouse was prepared from choroid tissue in (**A**). VEGF expression was determined by using qRT-PCR and compared between control and RBP-J cKO group. (**C**) CNV lesions of mice in (**A**) were sectioned and stained with anti-F4/80 and anti-VEGF, and observed under a confocal microscope. Five representative images from one eye were randomly selected and F4/80^+^VEGF^+^ cells were counted and compared in two groups (five eyes from five mice per group). (**D**) Flat mounted choroidal tissues in (**A**) were stained with anti-F4/80 and anti-TNF-α and observed under a confocal microscope. TNF-α immuno-reactivities were compared between control and RBP-J cKO group. (**E**) Total RNA of two eyes from one mouse was prepared from choroid tissue in (**A**). TNF-α expression was determined by using qRT-PCR and compared between control and RBP-J cKO group. (**F**) CNV lesions of mice in (**A**) were sectioned and stained with anti-F4/80 and anti-TNF-α, and observed under a confocal microscope. F4/80^+^TNF-α^+^ cells were compared. Five representative images from one eye were randomly selected and F4/80^+^ TNF-α^+^ cells were counted and compared in two groups (five eyes from five mice per group). Data are presented as mean ± SEM (n = five mice per group). *P < 0.05, **P < 0.01. NS, no significance.

We also examined the local expression of VEGF and TNF-α by macrophages in CNV lesion areas using quantitative RT-PCR and immunostaining (Fig. 6). The number of F4/80+VEGF+ macrophages in the lesion area of RBP-J cKO mice was less than that of control mice. Intriguingly, the total VEGF mRNA level in the eyecup of the two groups showed no significantly difference (Fig. 6A–C). The number of F4/80+TNF-α+ macrophages in the lesion area of RBP-J cKO mice was also less than that of control mice, while the TNF-α mRNA level in the eyecup of RBP-J cKO mice decreased consistently (Fig. 6D–F).

### Myeloid RBP-J Knockout Attenuated Endothelial Tube Formation

**Figure 7:**
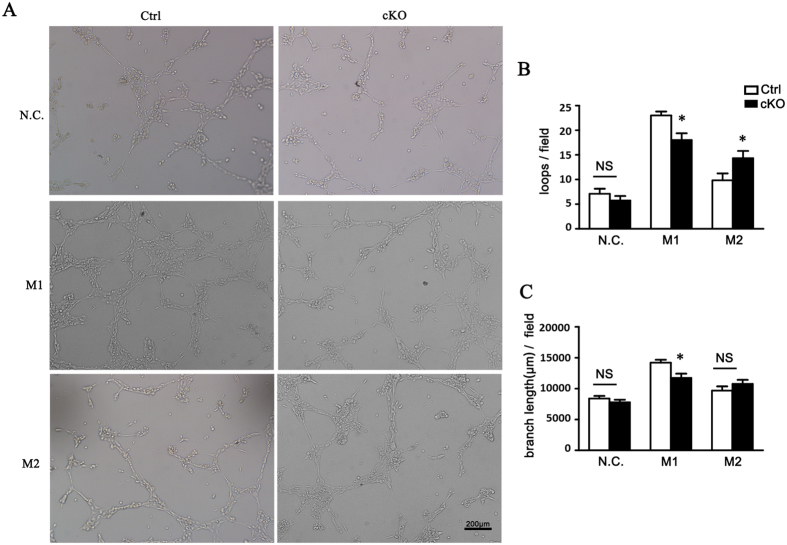
Reduced pro-angiogenic phenotype of RBP-J-deficient macrophages inhibited angiogenesis. (**A**) BMDMs were prepared from RBP-J cKO and control mice, and stimulated with PBS (N.C), LPS+IFN-γ (M1) or IL-4 (M2). bEND.3 cells were cultured in the presence of conditional medium (CM) from differentially activated macrophages, and induced to form lumens *in vitro*. Representative images of bEND.3 cells cultured with CM of N.C, M1 or M2 macrophages were shown. (**B**) Five representative microscopic fields were selected randomly in each culture condition, and the number of lumen loops was calculated and branch length of cell cords of the enclosed lumens was measured. The average number of lumen loops and the average branch length were calculated and compared. Data are presented as mean ± SEM, n = five individual experiments. *P < 0.05. NS, no significance.

To establish if attenuated pro-angiogenic effect of macrophages subsets from RBP-J cKO mice was caused by secreted growth factors, we performed a tube formation assay with conditioned media of different macrophage subsets derived from RBP-J cKO and the control groups (Fig. 7). Conditioned media of M1 and M2 macrophages all stimulated tube formation in the control group. However, tube formation was attenuated in conditioned medium of M1 from RBP-J cKO mice, while was enhanced in conditioned medium of M2 from RBP-J cKO mice (Fig. 7A). Both vascular loops and branch length were inhibited in M1 RBP-J cKO macrophages medium culture system, while only vascular loops were increased in M2 RBP-J cKO macrophages medium culture system (Fig. 7B,C). Therefore, in associated with the decreased M2 polarization in CNV lesions as demonstrated above, these results suggested that myeloid RBP-J deletion attenuated endothelial tube formation in CNV lesions, which was at least partially caused by the secreted cytokines.

## Discussion

It has been recognized that cells of the mononuclear-macrophage lineage are key cellular components of CNV lesions^29^,^30^. Several studies have demonstrated that, to sustain macrophage infiltration at sites of injury, circulating monocytes are recruited to the lesion area at the early stage of CNV and play a crucial role in the initial events of CNV^9^,^30^,^31^,^32^. Therefore, modulating macrophage recruitment and activation is a potential therapeutic strategy for CNV treatment. Notch signal plays an important role in inflammatory responses through macrophage activation in diseases such as Alzheimer’s disease, diabetic complications and chronic liver injuries^33^,^34^, and also has been shown to participate in CNV growth in different models^24^,^25^,^28^. As one of the critical signaling pathways regulating various aspects of development, the consequences of Notch signal intervention are strongly cell context-dependent. The myeloid specific RBP-J knockout mice model used in this study achieves a conditional disruption of Notch signaling in myeloid-macrophage lineage, and importantly provides a platform permitting an *in vivo* investigation of the function and behavior of macrophages in the context of CNV. In current study, we have demonstrated for the first time that modulation of macrophage phenotype and infiltration through Notch signaling can inhibit angiogenesis and reduce the extent of pathological fibrovascular formation in the eye.

The reduction of CNV lesion size in RBP-J cKO mice is a key finding of this study. Interestingly, by using the RBP-J conditional knockout mouse and Mx-Cre transgenic mouse in which Cre expression could be induced by poly(I)-poly(C), we previously have shown that when Notch signaling was globally disrupted, CNV growth was greatly promoted^25^,^35^. Also, in this inducible Mx-Cre-RBP-J conditional knockout mouse, we observed induced spontaneous angiogenesis in multiple tissues, including retina and cornea, which do not present in myeloid RBP-J cKO mice. The contrasting effects of global knockout and myeloid RBP-J knockout on CNV could be attributed to cell context-dependent consequences, as Notch signal is one of the critical signaling pathways regulating various aspects of development. Till now, the essential functions of the Notch signal in tip cell selection, endothelial quiescence and vessel maturation have been widely identified^26^. However, the precise mechanisms of Notch signaling are far from being fully illustrated at each stage of angiogenesis, as the process is orchestrated by complicated cellular interactions and cytokines. Global deficiency of Notch signaling is more likely to influence CNV through effects on other cell types such as endothelial cells, RPE cells, and other vessel-associated cells. Notch signal activation in macrophages has been previously reported under several pathological conditions^13^,^18^,^20^. Consistent with previous reports^28^, we showed Notch1 expression could be detected in early stage after CNV induction. In this study, we further observed that the active Notch1 intracellular domain was detected in macrophages after CNV induction at day 3, and increased up to the day 7. Particularly, increased active Notch signal and Notch downstream genes were observed in macrophages in CNV lesions 7 days after laser induction. By using a murine soluble Dll4 protein, Camelo *et al*. showed opposing effects of Dll4-Notch signal on vascular endothelium and macrophages, in which Dll4 activation accentuates the angiogenic potential of macrophages but inhibits neovascularization by its direct effect on endothelial cell proliferation^28^. Consistently, in our study, when Notch signal is deleted specifically in myeloid cells, CNV growth is inhibited instead of those findings that CNV was promoted in Dll4+/− mice. Therefore, the different outcomes between the two models in our research might be due to the functions of Notch signaling in different cell types involved in CNV growth, which also needs further investigations. These findings highlight the necessity to evaluate Notch activity of the different cellular involvement and in different biological settings individually.

It has been believed that macrophages play diverse roles in CNV^29^. There are two types of macrophages, tissue resident microglia and blood monocyte-derived macrophages, which have different developmental origin, infiltrating the lesion area after CNV. However, the latter one is assumed to account for the major ratio^30^,^36^,^37^. These macrophages could be activated and polarized into different functional states by environmental and intrinsic cues and play either a pro-inflammatory (M1) or a pro-angiogenic (M2) role in ocular angiogenesis including CNV. In CNV model, evidence has shown that sDLL4 activates Notch signaling and induces pro-angiogenic mediators in macrophages^28^, but its effect on the infiltration and polarization is still uncovered. We here show that less infiltration of macrophages in CNV lesion is observed in RBP-J cKO mice. We assume that there are at least two reasons to explain the phenomena. Firstly, Notch signaling might be required for initial macrophage activation and participate in polarization at later inflammatory stages. Therefore, blocking Notch signaling by genetic methods may result in a deficiency of initial macrophage activation, mobilization and lead to the absence of both M1 and M2 types of macrophages. We here do observe that both M1 and M2 macrophages are reduced in RBP-J cKO mice, and especially M2 are greatly lost. Secondly, various regulators exist in the CNV niche including growth factors, cytokines, chemokines, and extracellular matrix components. The activation/suppression of these factors indirectly resulted from Notch deficiency in macrophages might further aggravate the loss of macrophages in CNV.

He *et al*.^8^ demonstrated predominant M2 macrophages in the laser-induced CNV model. Consistently, we also observed more M2 macrophages existing in the sites in both RBP-J cKO mice and control mice. The question of how macrophages acquire specific M2 phenotypes in the CNV context remains to be answered. One notion is that infiltrating myeloid cells are polarized and conditioned by cytokines and certain signal pathways. Previous studies in our group showed that Notch signaling is instructive for M1 and suppressive for M2 polarization in microglia, tumor-associated macrophages and macrophages in spinal cord injury^16^,^22^,^38^. However, in the current study, with RBP-J deletion, M2 macrophages appear to decrease while M1 type remains in a moderate level. Therefore, the differences found in the inflammatory response with Notch deficiency may depend on the local inflammatory milieu and the type of inflammatory stimulators.

The decrease of M2 macrophages that appear after laser induction in RBP-J cKO mice might attribute to the attenuation of CNV growth. Evidence has shown that this pro-angiogenic potential of M2 macrophages may present directly or indirectly through various growth factors and cytokines. It has been reported that prior to angiogenesis of CNV, Arg-1+ macrophages recruited to the injury site demonstrate an enhanced VEGF expression. After laser induction, these recruited microglia/myeloid cells are the earliest source for VEGF production from day 2 to day 4, but almost lost till day 7 within the lesion, indicating early and transient contribution of myeloid cells-derived VEGF to CNV development^9^. VEGF derived from macrophages is more likely a trigger for retinal pigment epithelium (RPE) to secret VEGF further, of which the latter accounts for the main source of VEGF in CNV. Thus, it could explain the finding in our study that, when Notch signal is deleted in macrophages, the number of VEGF+ macrophages decreased significantly but the overall expression of VEGF in CNV lesions did not change remarkably. However, recent data suggested that targeted deletion of macrophages or RPE-derived VEGF expression does not alter CNV size, indicating that VEGF-independent mechanism(s) also exist to initiate choroidal angiogenesis^8^,^9^. Altered expression of the pro-angiogenic factor TNF-α in macrophages and in CNV sites was observed in our study, suggesting that TNF-α may contribute to VEGF-independent angiogenic responses. TNF-α has been shown to promote laser-induced CNV, and inhibition of TNF-α could reduce CNV development^30^,^39^,^40^. However, more evidence is required to pinpoint that M2 macrophage is the main source of TNF-α in CNV.

In summary, our results demonstrate that deletion of myeloid RBP-J/Notch signal inhibits early inflammatory response in the retina and choroid after injury, reduces Arg1+ monocyte recruitment to the choroid, and suppresses VEGF and TNF-α production and CNV development in the choroid. These findings support the notion that targeting macrophages and its related inflammation may be beneficial for treatment of patients with CNV, and have implications for the design of combination therapies. Furthermore, the results provide a rationale to further explore Notch signal pathway in distinct cell contexts as a novel therapy for patients with CNV and other intraocular inflammatory disorders.

## Materials and Methods

### Animals

Eight-week old male mice were used in our study. All wild-type C57/BL6 and transgenic mice were maintained under specific pathogen free conditions. The Lyz2-Cre transgenic (Jackson Laboratory, Bar Harbor, ME) and RBP-J-floxed (RBP-J^f^)^41^ mice were crossed to obtain RBP-J^f/+^ and RBP-J^f/f^ mice bearing the Lyz2-Cre transgene (hereafter referred to as control and RBP-J cKO, respectively), which were identified by polymerase chain reaction (PCR) with tail DNA as a template^41^. Primers for Lyz2-Cre: N1: 5′-CCGGTCGATGCAACGAGTGATGAGG; N2: 5′-GCCTCCAGCTTGCATGATCTCCGG; Primers for WT: R3: 5′-GTTCTTAACCTGTTGGTCGGAACC; R4: 5′-GCTTGAGGCTTGATGTTCTGTATTGC; Primers for RBP-J-floxed: R3: 5′-GTTCTTAACCTGTTGGTCGGAACC; PGKD1: 5′-ACCGGTGATGTGGAATGTGT. All animal experiments were approved by the Animal Experiment Administration Committee of Fourth Military Medical University. All animal manipulations were carried out in accordance with the National Institutes of Health Guide for the Care and Use of Laboratory Animals (NIH Publications, eighth edition) revised in 2012, and all efforts were made to minimize animal number and their suffering.

### CNV Induction

Laser photocoagulation was performed on the right eyes of mice using a 532 nm laser (Iris Radiation Systems, USA; spot size 75 μm, power100 mw, duration 0.1 s) through a contact lens. Six laser spots were applied between the major retinal vessels 1.5–2 disc diameters from the optical disc. Disruption of Bruch’s membrane was confirmed when a cavitation bubble appeared without hemorrhage.

### CNV Lesion Assessment

Choroidal flatmount was performed on day 3, 7 and 14 after CNV induction as described previously^25^. Anesthetized mice were perfused transcardially with 50 ml of phosphate-buffered saline (PBS) and 50 ml of 4% paraformaldehyde (PFA). Eye globes were enucleated and post-fixed for 4 h in 4% PFA, and then the anterior segment and the neural retina were removed. The remaining RPE-choroid-sclera complex was flatmounted with six radial cuts or more, and then permeabilized in 0.2% Triton X-100 for 24 h. Isolectin B4 (1:200, FL-1201, Vector) or primary antibodies in PBS containing 1% BSA and 0.05% Triton X-100 were incubated overnight at 4 °C, followed by incubation with secondary antibodies in PBS for 2 h at room temperature. Each step was followed by three washes in PBS for 10 min. Flatmounts were examined and photographed under a confocal laser scanning microscopy. Primary antibodies included rat anti-mouse F4/80 (1:400, 14-4801, ebioscience), rabbit anti-mouse VEGF (ab46154), rabbit anti-mouse TNF-α (ab6671) (1:200, Abcam). Secondary antibodies were goat anti-rat TRITC (1:400, CW-0167, Vector) and goat anti-rabbit IgG-CY3 (1:400, C2306, Sigma).

For CNV volume, horizontal optical sections (2 μm step) were obtained from the surface of the RPE-choroid-sclera complex. The deepest focal plane in which the surrounding choroidal vascular network connecting to the lesion could be identified was judged to be the floor of the lesion. Any vessels in the laser-treated area and superficial to this reference plane was judged as CNV. Images of each section were digitally stored. The area of CNV-related fluorescence was measured by computerized image analysis with the microscope software (TCS SP; Leica). The summation of whole fluorescent area in each horizontal section was used as an index for the volume of CNV.

### Histology and Immunofluorescence

Anesthetized mice were perfused and post-fixed as described earlier. The corneas and lens were removed to make the eye cups, which were then dehydrated in 30% sucrose overnight. Frozen sections were made by using a cryostat microtome (Leica, Nussloch, Germany). A series of 8-μm-thick sagittal sections were made and placed on gelatin-coated slides. Sections were then washed and blocked with PBS containing 1% bovine serum albumin (BSA) and 0.3% Triton X-100 for 0.5 h at room temperature. Slides were incubation with primary and secondary antibodies, and were then counterstained with 4′,6-diamidino-2-phenylindole (DAPI) (D8417, Sigma, St. Louis, MO) for 10 min at room temperature. Each step was followed by three washes in PBS for 10 min. Slides were closed with coverslips. The sections were examined and photographed under a confocal laser scanning microscopy. Primary antibodies included rat anti-mouse F4/80 (1:400, 14-4801, eBioscience), mouse anti-iNOS (1:500, ab49999, Abcam), goat anti-Arg-1 (1:500, ab60176, Abcam), rabbit anti-mouse VEGF(1:200, ab46154, Abcam), rabbit anti-mouse TNF-α (1:200, ab6671, Abcam), rabbit anti-mouse Notch1-ICD (1:800, ab8925, Abcam). Secondary antibodies were goat anti-rat IgG-biotin (BA-9400), Avdin Cy3 (SA-5549), Streptavidin FITC (SA-5488) (1:500, Vector), goat anti-rabbit IgG-Cy3 (1:400, C2306, Sigma), donkey anti-goat IgG (1:500, Alexa Flour 488, CA11055S, Invitrogen), and donkey anti-mouse IgG (1:500, Alexa Flour 488, CA21202S, Invitrogen).

For staining retinal blood vessels, the retina was separated and blocked overnight before it was stained with isolectin B4 as described earlier. Under a dissecting microscope, the retina was flat mounted on a glass slide and then covered. The retinas were photographed under a confocal laser scanning microscopy. Vessel density was measured by counting branch point number in randomly selected fields (1200 μm × 1200 μm, five fields per retina).

Eye cups of mice were obtained as described earlier. Tissues were then fixed in Bouin’s fixatives, embedded in paraffin, sectioned at 6 μm, and stained with hematoxylin and eosin (H&E) by standard methods.

### Transmission Electron Microscopy (TEM)

The mouse eyes were enucleated and fixed in 2.5% glutaraldehyde in cacodylate buffer at room temperature for 12 h, followed by post-fixation, dehydration, and embedding in Epon-araldite. Semi-thin sections (1 μm) through the optic nerve were prepared, stained with toluidine blue, and examined under a light microscopy. Ultra-thin section (0.5 μm) of selected areas was then prepared and double-stained with uranyl acetate and lead citrate for electron microscopy (CM-12 TEM; Philips). We used at least ten digital images for each sample to measure the thickness of Bruch’s membrane at a magnification of x20,000. A transparent grid was superimposed onto the micrograph, with RPE basement membrane aligned with the horizontal line. Five random measurements were made on each digital image using Image J software.

### Macrophage Culture

Bone marrow (BM) was flushed from femurs and tibias. BM-derived macrophages (BMDMs) (2 × 10^6^) were cultured in 12-well plates in 1 mL of α-MEM medium containing 10% FCS, 2 mmol/L L-glutamine, and 40 ng/mL murine granulocyte macrophage colony stimulating factor (GM-CSF, Pepro Tech, Inc.) for the indicated periods of time. LPS (100 ng/mL), IFN-γ (20 ng/mL) or IL-4 (20 ng/mL, Sigma) was added to culture medium 24 h before the end of the culture. A γ-secretase inhibitor IX (GSI; Calbiochem) was added at the concentration of 75 μmol/L with DMSO as a control.

### Flow Cytometry

Enucleated eyes were prepared under a dissection microscope on day 3 after CNV induction, and RPE/choroid were separated. For isolation of RPE/choroidal cells, tissue was digested in Hank’s balance salt solution (PAA) containing 0.5 mg/ml collagenase IV for 30 min at 37 °C. The cell suspensions were then passed through a cell strainer, washed and resuspended in staining buffer. Single-cell suspensions from pooled RPE/choroidal samples from two eyes were then blocked with 10% rat serum, before incubation with FITC-F4/80 (1:100, 11-4801, eBioscience) and PE-CD11b (1:400, 55-7397, eBioscience) for 30 min at 4 °C. Fluorescence-activated cell sorter (FACS) analysis was performed using a FACSCalibur^TM^ (BD Immunocytometry Systems). Data were analyzed with the FlowJo vX.0.6 software (FlowJo, LLC, Ashland, OR). Dead cells were excluded by propidium iodide (PI) staining.

### Quantitative Reverse Transcription (RT)-PCR (qRT-PCR)

Total RNA of choroids or macrophages was extracted by using the TRIzol reagent (Invitrogen) according to the manufacturer’s instructions. cDNA was prepared by using a reverse transcription (RT) system (Takara Dalian, Dalian, China). Quantitative real-time PCR was performed in triplicates by using a kit (SYBR Premix EX Taq; Takara) and the ABI PRISM 7500 Real-Time PCR System, with β-actin as an internal control. The PCR primers are listed in Table 1.

### Enzyme-Linked Immunosorbent Assay (ELISA)

The concentrations of TNF-α, IL-1β, IL-6, IL-10 and VEGF were measured by using ELISA with kits (Neobioscience) following manufacturer’s protocol.

### Endothelial Lumen Formation Assay

Matrigel Basement Membrane Matrix (BD Biosciences) was thawed overnight at 4 °C and diluted with medium to coat wells of 48-well dishes at 37 °C for 40 min. Brain Microvascular bEND.3 endothelial cells (1 × 10^5^) were seeded on the gel in 400 μl of medium and incubated at 37 °C and 5% CO_2_ for 6 h. Images were captured by using a converted microscope with a CCD camera. The network formation was quantified by counting branches and the length of the enclosed lumens.

### Statistics

Images were processed using the Image ProPlus 5.1 software (Media Cybernetics, Bethesda MD). Data were analyzed with GraphPad Prism 5 software (San Diego, CA), v. 5.0. Student t test or paired t-test was used for the statistical analyses. P < 0.05 was considered statistically significant.

## Additional Information

**How to cite this article**: Dou, G.-R. *et al*. Myeloid-Specific Blockade of Notch Signaling Attenuates Choroidal Neovascularization through Compromised Macrophage Infiltration and Polarization in Mice. *Sci. Rep.* **6**, 28617; doi: 10.1038/srep28617 (2016).

## Figures and Tables

**Table 1:** Primers used in the study.

Gene	Sequence
*Actin*	F:5′-CAT CCG TAA AGA CCT CTA TGC CAA C
*Actin*	R:5′-ATG GAG CCA CCG ATC CAC A
*Arg1*	F:5′-AGA CAG CAG AGG AGG TGA AGA G
*Arg1*	R:5′-CGA AGC AAG CCA AGG TTA AAG C
*Hes-1*	F:5′-GCA GAC ATT CTG GAA ATG ACT GTG A
*Hes-1*	R:5′-GAG TGC GCA CCT CGG TGT TA
*Hey-1*	F:5′-CAT GAA GAG AGC TCA CCC AGA
*Hey-1*	R:5′-CGC CGA ACT CAA GTT TCC
*iNOS*	F:5′-GCA GAG ATT GGA GGC CTT GTG
*iNOS*	R:5′-GGG TTG TTG CTG AAC TTC CAG TC
*IL-1β*	F:5′-TCCAGGATGAGGACATGAGCAC
*IL-1β*	R:5′-GAACGTCACACACCAGCAGGTTA
*IL-6*	F:5′-CCA CTT CAC AAG TCG GAG GCT TA
*IL-6*	R:5′-GCA AGT GCA TCA TCG TTG TTC ATA C
*IL-10*	F:5′-CCC TTT GCT ATG GTG TCC TT
*IL-10*	R:5′-TGG TTT CTC TTC CCA AGA CC
*MR F*	F:5′-AAA CAC AGA CTG ACC CTT CCC
*MR R*	R:5′-GTT AGT GTA CCG CAC CCT CC
*TNF-α*	F:5′-CAG GAG GGA GAA CAG AAA CTC CA
*TNF-α*	R:5′-CCT GGT TGG CTG CTT GCT T
*VEGFA*	F:5′-AGG AGC CGA GCT CAT GGA
*VEGFA*	R:5′-CTC TCC TTC TGT CGT GGG TG
